# Changes in Childhood Pneumonia Hospitalizations by Race and Sex Associated with Pneumococcal Conjugate Vaccines

**DOI:** 10.3201/eid2206.152023

**Published:** 2016-06

**Authors:** Andrew D. Wiese, Carlos G. Grijalva, Yuwei Zhu, Edward F. Mitchel, Marie R. Griffin

**Affiliations:** Vanderbilt University Medical Center, Nashville, Tennessee, USA (A.D. Wiese, C.G. Grijalva, Y. Zhu, E.F. Mitchel Jr, M.R. Griffin);; VA Tennessee Valley Healthcare System, Nashville (C.G. Grijalva, M.R. Griffin)

**Keywords:** pneumococcal conjugate vaccine, 7-valent pneumococcal conjugate vaccine, PCV7, 13-valent pneumococcal conjugate vaccine, PCV13, pneumonia, invasive pneumococcal disease, pneumococci, bacteria, health status disparities, children, hospitalization, race, sex, vaccines, Tennessee, United States

## Abstract

Introduction of pneumococcal conjugate vaccines in the childhood immunization schedule was associated with decreases in all-cause pneumonia hospitalizations among black and white children in Tennessee, USA. Although racial disparities that existed before introduction of these vaccines have been substantially reduced, rates remain higher in boys than in girls among young children.

Introduction of the 7-valent pneumococcal conjugate vaccine (PCV7) into the childhood immunization schedule was associated with substantial reductions in invasive pneumococcal disease (IPD) and pneumonia in young children in the United States and elsewhere (*1*–*4*). In the United States specifically, black children traditionally had higher IPD and pneumonia rates than white children. However, this racial disparity in IPD was reduced after introduction of PCV7 (*5*).

After the introduction of the 13-valent pneumococcal conjugate vaccine (PCV13) in 2010, IPD and pneumonia hospitalizations among children <5 years of age were reduced further, and racial differences in IPD reached an historic low (*2*,*6*,*7*). However, the effect of PCVs on racial disparities for pneumonia, which is much more common than IPD, remains unclear. Furthermore, sex seems to persist as an independent risk factor for IPD after PCV7 introduction (*8*).

Identifying high-risk groups is essential for planning preventative strategies, but few studies have directly examined sex differences in pneumonia incidence since introduction of PCVs (*9*). Therefore, we examined changes in childhood pneumonia hospitalization rates between racial and sex groups since introduction of PCVs.

## The Study

We used hospitalization data from all nonfederal hospitals in Tennessee for 1998–2013 in the Tennessee Hospital Discharge Data system. Pneumonia hospitalizations among Tennessee residents <18 years of age were defined by an International Classification of Diseases, Ninth Revision, Clinical Modification, coded primary discharge diagnosis of pneumonia, or any diagnosis of pneumonia with a primary diagnosis of meningitis, septicemia, or empyema (*10*,*11*). The strategy of using this classification for identification of pneumonia hospitalizations has been validated in other data systems (*10**,**11*). We also analyzed the rate of hospitalizations with a primary discharge diagnosis of pneumonia only. 

Racial categories were black and white. Race-specific rates were not calculated for Hispanic and other racial-ethnic categories because of low numbers of hospitalizations (7.9% of total). Age groups were <2, 2–4, and 5–17 years of age. Four periods were defined that excluded years of vaccine introduction: pre-PCV7 (1998–1999), early PCV7 (2001–2005), late PCV7 (2006–2009), and post-PCV13 (2011–2013).

Annualized period rates were calculated for each study period by dividing the average annual pneumonia hospitalizations by the average annual Tennessee population estimate for all children combined and grouped by age, sex, and race (US Census Bureau and the National Center for Health Statistics Bridged Race Population Estimates). Rates were expressed as pneumonia hospitalizations/1,000 children annually. For comparisons, rate ratios and rate differences were calculated with 95% CIs to assess significance of the estimates at the 5% level. All analyses were performed by using STATA version 14 (StataCorp LP, College Station, TX, USA).

Pneumonia hospitalization rates decreased among children <18 years of age during the study period. Overall and age-specific pneumonia hospitalization rates include all race/ethnicity categories (black, white, Hispanic, other) because these estimates were not different from estimates that included only black and white children (Table). Most hospitalizations were for children <2 years of age in the pre-PCV7 period (49.0%) and for children 5–17 years of age in the post-PCV13 period (38.4%).

**Table:** Pre-PCV7 and post-PCV13 annualized pneumonia rates, by age, sex, and race, and hospitalizations per 1,000 children, Tennessee, USA, 1998–2013*

Patient age, y, and race	Patient sex	Hospitalizations/1,000 children			Post-PCV13 vs. pre-PCV7	
		Pre-PCV7, 1998–1999, 11,059 hospitalizations	Post-PCV13, 2011–2013, 9,171 hospitalizations		Rate ratio (95% CI)	Rate difference (95% CI)†
All‡		4.0	2.0		0.51 (0.50–0.52)	−2.0 (−2.1 to −1.9)
<2‡		18.4	7.3		0.40 (0.38–0.41)	−11.1 (−11.6 to −10.6)
Black	M	27.1	8.5		0.31 (0.28–0.35)	−18.6 (−20.5 to −16.6)
Black	F	20.0	6.4		0.32 (0.28–0.37)	−13.6 (−15.3 to −11.9)
White	M	18.0	7.8		0.43 (0.40–0.46)	−10.2 (−11.1 to −9.3)
White	F	14.5	6.2		0.43 (0.40–0.47)	−8.2 (−9.0 to −7.4)
2–4‡		5.4	3.0		0.56 (0.52–0.59)	−2.4 (−2.6 to −2.1)
Black	M	6.6	3.4		0.50 (0.43–0.59)	−3.3 (−4.1 to −2.5)
Black	F	5.3	3.1		0.58 (0.49–0.70)	−2.2 (−3.0 to −1.5)
White	M	5.3	3.0		0.56 (0.51–0.62)	−2.3 (−2.7 to −1.9)
White	F	5.0	2.9		0.59 (0.53–0.65)	−2.0 (−2.4 to −1.6)
5–17‡		1.6	1.1		0.67 (0.63–0.70)	−0.5 (−0.6 to −0.5)
Black	M	2.0	1.1		0.57 (0.49–0.65)	−0.8 (−1.1 to −0.6)
Black	F	1.6	1.0		0.61 (0.53–0.71)	−0.6 (−0.8 to −0.4)
White	M	1.6	1.1		0.70 (0.65–0.75)	−0.5 (−0.6 to −0.4)
White	F	1.6	1.2		0.74 (0.68–0.80)	−0.4 (−0.5 to −0.3)

*PCV7, 7-valent pneumococcal conjugate vaccine; PCV13, 13-valent pneumococcal conjugate vaccine. †For hospitalizations per 1,000 children. ‡Includes all race/ethnicity categories (black, white, Hispanic, other). These estimates were not different from estimates including only black and  white children.

Overall, rates of pneumonia hospitalizations were higher among black children than among white children in the pre-PCV7 period for boys and girls (rate ratio [RR] 1.37, 95% CI 1.29–1.45 and RR 1.19, 95% CI 1.12–1.27, respectively). However, during the post-PCV13 period, black and white girls showed similar rates of pneumonia hospitalizations (RR 0.98, 95% CI 0.91–1.06). Rates for black boys also decreased but were still higher than rates for white boys (RR 1.12, 95% CI 1.05–1.20) during the post-PCV13 period.

For children <2 years of age, pneumonia hospitalization rates decreased in each successive period for each of the 4 race/sex groups (Figure). Among children <2 years of age, black boys, black girls, and white boys showed higher pneumonia rates than white girls during the pre-PCV7 and PCV7 periods. In the post-PCV13 period, black and white girls showed similar rates, and rates for black and white boys remained higher than rates for white girls among children <2 years of age. Decreases for each of the 4 race/sex groups were also observed for children 2–4 and 5–17 years of age (Figure). However, no disparities were observed by race or sex in the post-PCV13 period for children 2–4 and 5–17 years of age (Figure).

**Figure:**
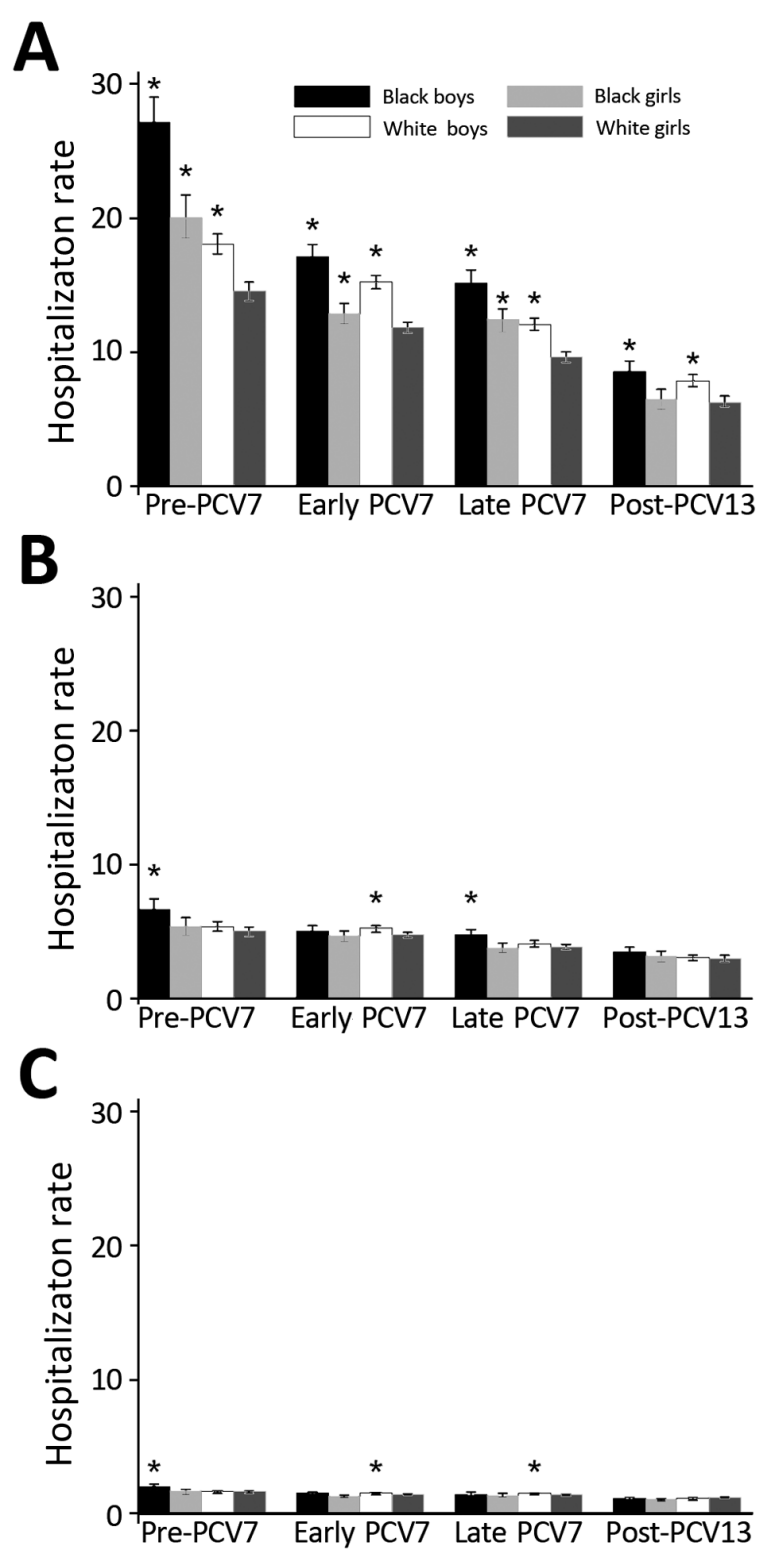
Annualized all-cause pneumonia hospitalization rates, by age group, race, and sex, per 1,000 children, Tennessee, USA, 1998–2013. A) <2 years of age; B) 2–4 years of age; C) 5–17 years of age. Asterisks indicate groups with a significantly higher rate than white girls within each period, determined on the basis of the 95% CI of the respective rate ratio (not shown). Error bars indicate 95% CIs of hospitalization rate. Periods were defined as follows: pre-PCV7, 1998–1999; early PCV7, 2001–2005; late PCV7, 2006–2009; and post-PCV13, 2011–2013.

When we compared post-PCV13 with pre-PCV7 for all age groups, we found that black boys had the greatest relative and absolute decreases for pneumonia hospitalization rates; the largest decreases were for children <2 years of age. We estimated that there were 18.6 fewer pneumonia hospitalizations per 1,000 black boys <2 years of age during the post-PCV13 period than during the pre-PCV7 period (Table). Similar trends were observed when we restricted our assessment to the subset of hospitalizations with a primary discharge diagnosis of pneumonia.

## Conclusions

Rates of pneumonia hospitalizations decreased among black and white children in Tennessee after introduction of PCVs, in particular among children <2 years, with only minimal residual racial differences in the post-PCV13 period. In spite of these reductions, for children <2 years of age, rates of pneumonia hospitalizations for black and white boys were consistently higher than for girls. However, no differences were observed by race or sex in the post-PCV13 period for children 2–4 and 5–17 years of age.

An explanation for the persistent differences by sex in pneumonia incidence could be the proposed effect of sex hormones on the immune response or differences in behavior among young boys and girls (*9*,*12*). However, these observations require further scrutiny because few studies have examined the association between infection risk and sex-associated behavior or immune response in children (*9*,*12*).

Our study had several limitations. First, this was an ecologic study, and we cannot attribute changes in pneumonia hospitalization directly to PCVs. However, these designs are preferred for the evaluation of programs with high uptake and potential indirect benefits, such as PCV vaccination programs, because they avoid known selection issues that could affect comparisons of vaccinated and unvaccinated persons, and estimates of effect include those resulting from indirect vaccine effects. Second, the study was restricted to only hospitalizations, and additional studies conducted in the outpatient setting would be useful to complement these observations. However, previous studies have documented no compensatory increases in emergency department visits that could result from observed reductions in hospitalizations (*13*). Moreover, another study reported no changes in hospitalizations for fractures among children in Tennessee during 1998–2012 associated with introduction of PCVs, which should reduce concerns about generalized changes in hospitalization practices in Tennessee (*2*). Third, other factors, such as socioeconomic characteristics or concurrent conditions, could contribute to observed differences between racial and sex groups. However, our study did not account for those factors. Fourth, because our study documents changes for only children in Tennessee, caution is warranted when extrapolating our findings to other settings.

Overall, these findings suggest that PCV7 and PCV13 have substantially reduced racial disparities in all-cause pneumonia between black and white children in Tennessee that existed before PCVs, especially among children <2 years of age. Although relative rate comparisons by sex for children <2 years of age after introduction of PCV13 indicated statistically significant differences, the absolute differences in rates were small and much lower than in the pre-PCV7 period.
