# Correction: Vol. 22, No. 6

**DOI:** 10.3201/eid2210.C42210

**Published:** 2016-10

**Keywords:** errata, erratum, correction

The descriptions of pneumonia diagnoses and hospitalizations were unclear in Changes in Childhood Pneumonia Hospitalizations by Race and Sex Associated with Pneumococcal Conjugate Vaccines (A.D. Wiese al.). The article has been corrected online (http://wwwnc.cdc.gov/eid/article/22/6/15-2023_article).

